# The Case of Dr. Spearman

**Published:** 1888-05

**Authors:** 


### THE CASE OF DR. SPEARMAN.

We regard the above case, found in the original department of this number of the RECORD, as one of *lumbo sacral neuralgia*, starting in a mechanical injury superinduced upon a neurotic deathesis.

The original injury probably caused a tearing or disarrangement of fibres, and led to a chronic thickening of the intervertebral ligaments, causing when in the erect, and in other positions, compression or pinching of a spinal nerve. The oft recuring pain has led to a neuritis which extending to the crural plexus affects by reflex irritation the great sciatic nerve and its distributions in the lower extremities.

Touching the treatment we may not suggest anything which our afflicted brother has not already tried. It would seem that benefit ought to result from rest in the recumbent position and a seaton, or a succession of blisters during a long period, over the lumbar region, followed at such times as pain is present by hypodermic injections of antipyrine or cocaine over the seat of suffering, and afterward by gentle currents of electricity.

As a tonic and antidyspeptic the following pill is suggested accompanied with such mild and nutritious diet as the stomach would best bear:

℞. Pyro. phos. iron........................................

Cinc ionidia........................................aa grs lx,

Strychnia........................................1 gr

Solid ex. valerian........................................q. s.

M. Make 60 pills. One to be taken three times a day.

We reported Dr. Spearman's case to the Medical Association of Georgia at its late meeting with our own views as above expressed, and asked the opinion of the members thereon. Nothing was elicited save an endorsement by several members, of our views. Dr. Holmes suggested massage with rest and hot salt water applications often repeated, and long continued, and seemed to regard the case as a hopeful one.

